# Prevalence of *Shigella* Serogroups and Their Antimicrobial Resistance Patterns in Southern Trinidad

**DOI:** 10.3329/jhpn.v26i4.1889

**Published:** 2008-12

**Authors:** Fitzroy A. Orrett

**Affiliations:** Department of Paraclinical Sciences, Unit of Pathology and Microbiology, Faculty of Medical Sciences, University of the West Indies, St. Augustine, Trinidad and Tobago

**Keywords:** Antibiotics, Community-based studies, Drug resistance, Microbial, *Shigella*, Dysentary, Bacillary, Trinidad and Tobago

## Abstract

The serogroup distribution and antimicrobial susceptibility patterns of *Shigella* isolates obtained from stool specimens of persons with acute diarrhoea in community-based studies from southern Trinidad during 1997-2006 were reviewed. Of the 5,187 stool specimens, 392 (8%) were positive for *Shigella* organisms. From these 392 isolates, 88.8% were recovered from children aged >0-10 year(s). *Shigella sonnei* was the most frequently-isolated serogroup (75%), followed by *S. flexneri* (19%), *S. boydii* (4.1%), and *S. dysenteriae* (1.8%). *S. flexneri* was the major isolate among the >20-30 years age-group. The most common drug resistance among all age-groups was to ampicillin. All strains of *S. flexneri, S. boydii,* and *S. dysenteriae* were fully susceptible to aztreonam, gentamicin, and ciprofloxacin. *S. sonnei,* the most common species isolated, showed resistance to all antibiotics tested. The data showed that, throughout the study period, the resistance to commonly-used drugs was relatively low. Since resistance to several drugs seems to be emerging, continuous monitoring of resistance patterns is mandatory for the appropriate selection of empiric antimicrobial drugs in the therapy of suspected cases of shigellosis.

## INTRODUCTION

Acute diarrhoeal diseases, a major public-health problem in developing countries, are often associa-ted with significant morbidity and mortality, especially among children (1-4). Of the pathogens causing diarrhoea, *Shigella* continues to play a major role in aetiology of inflammatory diarrhoea and dysentery, thus presenting a serious challenge to public-health authorities worldwide (5-7). A recent epidemiological report by Niyogi in 2005 (8) concluded that annually an estimated 165 million children and young adults worldwide suffer from shigellosis and that 99% occur in developing countries, and in developing countries, 69% of cases occur in children aged less then five years (9). These organisms belong to the Enterobacteriaceae family, with four serogroups described: *Shigella dysenteriae, S. flexneri, S. sonnei, and S. boydii. S. dysenteriae*, first described by Kiyoshi Shiga in 1897 (10), was a major cause of mortality during World War I, but decreased in prevalence after the war and was rapidly replaced by *S. flexneri* as the major serogroup, which caused a broader spectrum of diarrhoeal illnesses ranging from mild to very severe (6). After World War II, *S. sonnei* replaced *S. flexneri* as the dominant pathogen in most developed and some developing countries (6,11-13). *Shigella* species have been found in most surface-waters, sewage, food, and crops contaminated by human faeces used as fertili-zer (14,15). Although recovered from these sources, *Shigella* species are most frequently transmitted via direct person-to-person contact, and 10-100 organisms are required to start an infection (16).

In the late 1960s, *S. dysenteriae* type 1 was responsible for an epidemic in Guatemala, resulting in significant morbidity and mortality in that South American country (17). Subsequent to this outbreak, *S. dysenteriae* appeared in Asia and Africa and is now endemic in these regions (18-20).

Effective antimicrobial treatment for shigellosis has been shown to reduce its duration and severity, reduce shedding of the organisms and prevent potentially lethal complications (4). However, due to the global emergence of drug-resistance, the choice of antimicrobial agents for treating shigellosis is limited (21). Over the past 15 years, *Shigellae* have become progressively resistant to most widely-used and inexpensive antimicrobials, and changes in the incidence of these organisms from time to time have resulted in some challenges in formulating a drug of choice for therapeutic management of shigellosis. In Taiwan, 128 strains of *Shigella* tested against 11 antimicrobials were resistant to ampicillin (52%), chloramphenicol (84%), streptomycin (84%), and tetracycline (88%) (22). Reports from Indonesia (6), Bangladesh (23), Malaysia (5), and Nepal (24) showed increasing frequency of *Shigella* with multiple resistance to ampicillin, trimethoprim-sulphamethoxazole, tetracycline, and nalidixic acid. Similar resistance profiles were reported from Africa (19), Central America (17), Europe (25-27), and South America (28-29). Although antimicrobial resistance among *Shigella* species is well-documented in many countries, there is a lack of such documentation in Trinidad. The study was, therefore, undertaken to determine the frequency and serogroup distribution of *Shigella* from stools of patients with acute diarrhoea and to understand their resistant profiles to commonly-used antimicrobials, thus allowing for more appropriate management of shigellosis.

## MATERIALS AND METHODS

### Study area and population

During 1 January 1997*–*31 December 2006, stool specimens from outpatients who presented at the Accident and Emergency Department of the San Fernando General Hospital (SFGH), health centres, outpatient clinics at the SFGH, and offices of general practitioners with acute diarrhoea were received in the microbiology laboratory of the SFGH for analysis according to the standard methods (30). These patients came from both rural and urban areas. It was not possible to definitely assign any patient solely to either urban or rural areas because patients have relatives from both the areas and have used their addresses interchangeably. The SFGH is a 650-bed tertiary hospital located in the southern part of Trinidad. Trinidad is the larger island of the twin-island Republic—Trinidad and Tobago—located about 11 km off the northern coast of Venezuela in South America. The population of the Republic is about 1.3 million, and the SFGH serves a population of about 400,000. Specimens were transported in wide-mouth screw-on-top sterile containers and were processed within two hours of arrival in the laboratory.

### Bacteriological analysis

Stool specimens were primarily inoculated directly onto MacConkey agar and xylose-lysine deoxycholate (XLD) agar. All plates were incubated aerobically at 35-37° C for 18-24 hours. Lactose-non-fermenting colonies, morphologically resembling *Shigella*, were picked and again subcultured onto MacConkey and XLD media and were further identified biochemically using urea, triple sugar iron, sulphide-indole motility medium, and Simmons citrate media. Biochemically-screened strains of *Shigella* were further identified serologically to species level using polyvalent and monovalent antisera by the Caribbean Epidemiological Center (CAREC). CAREC, the regional branch of Pan American Health Organization/World Health Organization, is the reference laboratory for 19 Caribbean countries, including Trinidad and Tobago.

### Antimicrobial susceptibility testing

Antimicrobial susceptibility testing was performed by the disc-diffusion method using the guidelines and interpretative criteria of the Clinical Laboratory Standards Institute (CLSI) (30), with the following antimicrobials and concentrations: ampicillin (10 μg), tetracycline (30 μg), trimethoprim-sulphamethoxazole (1.25/23.75 μg), amoxicillin-clavulanic acid (20/10 μg), cefuroxime (30 μg), chloramphenicol (30 μg), ciprofloxacin (5 μg), aztreonam (30 μg), and gentamicin (10 μg) (Oxoid, UK). Multidrug resistance was defined as resistance to three or more antimicrobials. The control organism was *Escherichia coli* ATCC 25922 strain obtained from the CAREC.

Statistical analysis was performed using the SPSS software (version 12) (SPSS Inc., Chicago, Ill, USA), where prevalence was compared using chi-square.

## RESULTS

During the study period, 392 *Shigella* organisms were recovered from 5,187 stool specimens. The yearly distribution of these organisms is shown in Table 1. The number of stool specimens submitted each year to the laboratory remained relatively stable over the 10-year period. The predominant serogroup was *S. sonnei* which accounted for 75% (294/392) of all isolates, followed by *S. flexneri* (19%; 75/392), *S. boydii,* (4.1%; 16/392), and *S. dysenteriae* (1.8%; 7/392). The recovery of *S. sonnei* decreased from 44 (11.0%) isolates in 1997 to 26 (4.3%) isolates in 2006, and this was statistically significant (p<0.001). A similar pattern was observed for *S. flexneri* which decreased from 17 (4.3 %) in 1997 to 5 (0.8%) in 2006 (p<0.001). The isolation rates of *S. boydii* and *S. dysenteriae* remained relatively stable during the entire period.

**Table 1.** Yearly distribution and infection of *Shigella* serogroups from stool specimens of patients with diarrhoea at San Fernando General Hospital, 1997-2006

Year	Total no. of stool specimens	*S. sonnei* No. (%) isolated	*S. flexneri* No. (%) isolated	*S. boydii* No. (%) isolated	*S. dysenteriae* No. (%) isolated	Total No. (%) isolated
1997	399	44 (11)	17 (4.3)	2 (0.5)	0	63 (16)
1998	570	52 (9)	9 (1.6)	3 (0.5)	1 (0.2)	65 (11)
1999	713	43 (6)	13 (1.8)	3 (0.4)	0	59 (8)
2000	498	18 (3.6)	5 (1.0)	2 (0.4)	1 (0.2)	26 (5)
2001	489	15 (3.1)	8 (1.6)	1 (0.2)	0	24 (4.9)
2002	427	23 (5)	6 (1.4)	0	1 (0.2)	30 (7)
2003	467	17 (3.6)	6 (1.3)	1 (0.2)	1 (0.2)	25 (5)
2004	533	23 (4.3)	5 (0.9)	1 (0.2)	1 (0.2)	30 (6)
2005	487	33 (7)	1 (0.2)	1 (0.2)	0	35 (7)
2006	604	26 (4.3)	5 (0.8)	2 (0.3)	2 (0.3)	35 (6)
Total	5,187	294 (6)	75 (1.4)	16 (0.3)	7 (0.1)	392 (8)

There were significant falls in isolation (p<0.001) for both *S. sonnei* (44 to 26) and *S. flexneri* (17 to 5) for 1997 vs 2006

Table 2 shows the distribution of *Shigella* according to age-group. *Shigella* was isolated most frequently from the >0-10 years age-group. Almost 89% (348/392) of the isolates were recovered from stool specimens of this age-group, and the predominant serogroup was *S. sonnei* which accounted for 81% (n=280) of the 348 isolates. Also, among this age-group, *S. flexneri* was the next major serogroup isolated, accounting for 65% of all *S. flexneri* strains in this study. Among the >20-30 years age-group, *S. flexneri* was the most frequent serogroup recovered from diarrhoeal stool. The other serogroups—*S. boydii* and *S. dysenteriae*—were infrequently recovered from patients. Overall, *S. sonnei* and *S. flexneri* were responsible for 94% (369/392) of all the cases of shigellosis, and the former is the predominant serogroup in Trinidad.

**Table 2.** Distribution of *Shigella* cases according to age-group of patients with diarrhoea at San Fernando General hospital, 1997-2006

Age-group (years)	Total no. of stool specimens	*S. sonnei* No. (%)isolated	*S. flexneri* No. (%) isolated	*S. boydii* No. (%)isolated	*S. dysenteriae* No. (%)isolated	Total Shigella No. (%)isolated
0-10	4,876	280 (15)	49 (1.0)	13 (0.3)	6 (0.1)	348 (16)
>10-20	74	7 (12)	6 (6)	1 (1.1)	0	14 (19)
>20-30	60	2 (3.3)	16 (27)	0	1 (1.7)	19 (32)
>30-40	39	2 (5)	1 (2.6)	0	0	3 (8)
>40-50	39	1 (2.6)	0	0	0	1 (2.6)
>50	99	2 (2.0)	3 (3.0)	2 (2.0)	0	7 (7.0)
Total	5,187	294 (15)	75 (1.4)	16 (0.3)	7 (0.1)	392 (8)

The resistance rates of *Shigella* serogroups during the study period are shown in Table 3. Of the 392 *Shigella* isolates which included the four serogroups, only *S. sonnei* serogroup showed resistance to all nine antimicrobials. Among all serogroups, the most common resistance was to ampicillin. *S. boydii* and *S. sonnei* were more frequently resistant to tetracycline (37% and 36% respectively) than the other serogroups. All *S. flexneri, S. boydii* and *S. dysenteriae* serogroups were fully sensitive to aztreo-nam, gentamicin, and ciprofloxacin. Although varying degrees of resistance were noted to most drugs, susceptibility ranged from 52% to 100% for the most common serogroups recovered.

**Table 3.** Percentage of *Shigella* serogroups resistant to various antimicrobials studied at San Fernando General Hospital, 1997-2006

Antimicrobial	*Shigella* serogroups				Total (n=394)
	*S. sonnei* (n=294)	*S. flexneri* (n=75)	*S. boydii* (n=16)	*S. dysenteriae* (n=7)	
Tetracycline	36	12.0	38	0	32
Co-trimoxazole*	33	21	19	0	30
Ampicillin	9	48	63	100	16
Augmentin†	1.4	9	19	57	3.0
Cefuroxime	1.4	9	19	57	3.0
Chloramphenicol	1.4	0	19	0	1.5
Aztreonam	1.4	0	0	0	1.0
Gentamicin	1.4	0	0	0	1.0
Ciprofloxacin	0.7	0	0	0	0.5

*Trimethoprim-sulphamethoxazole;

†Amoxicillin-clavulanic acid

## DISCUSSION

*Shigella* was recovered from 8% of cases of acute infectious diarrhoea during the study period. This rate is comparable with studies from Israel (31), Indonesia (6), Nepal (32), and Ghana (33) that documented rates of 3.3%, 3.8%, 4.0%, and 5% respectively, but differed from higher rates reported from Bangladesh (58%) (23), Uganda (35%) (34), and Ethiopia (20%) (35). The low rate of isolation as observed in the present study maybe due, in part, to continuing educational programmes at elementary schools, aggressive infection-control measures in our hospital and healthcare centres, and possibly under-reporting of shigellosis cases by general practitioners. Shigellosis is primarily a childhood disease in both developed and developing countries whereas epidemic of shigellosis affects all age-groups (36). In this study, shigellosis was observed in all age-groups but was the highest among the >0-10 years age-group. Children within this age-group are most susceptible to shigellosis primarily because of poor resistance, lack of previous exposure, poor personal hygiene, and higher exposure to contaminated environment due to play-related activities (18,37).

All four serogroups of *Shigella* co-exist in different proportions in many countries. However, in most developing countries, *S. flexneri* is the predominant *Shigella* serogroup isolated from patients with infectious diarrhoea and represents 50-90% of all *Shigella* isolates (5,24,36,37). Our findings are in sharp contrast to these reports and agree with others from several developing countries where the predominant serogroup is *S. sonnei,* followed by S*. flexneri* (11,38-40). The other serogroups—*S. boydii* and *S. dysenteriae*—were infrequently isolated. The predominance of *S sonnei* did not change since the last decade as was evident in two previous reports from this country (7,41). This predominance was unlike the situation in the islands of Bengal (42) where *S. flexneri* and *S. dysenteriae* alternated as the most active agents of shigellosis.

Antimicrobial therapy is the cornerstone of treatment of shigellosis. The guiding principle for the choice of antimicrobial in developing countries includes the cost, availability of the drug, and the patterns of resistance in the community (21). The prevalence of resistance for the most prevalent serogroup—*S. sonnei*—during the study period ranged from 0.7% for ciprofloxacin to 36% for tetracycline. The prevalence of resistance was 1.4% for augmentin, cefuroxime, chloramphenicol, aztreonam, and gentamicin. Gram-negative bacterial resistance to tetracycline has always been variable and ranged from 6% to 78% (7,43-45). Antimicrobials are recommended for shigellosis because antimicrobials shorten the severity and duration of illness, reduce shedding of the organisms, and prevent subsequent infection by family contacts, development of secondary complications, and death. Antimicrobial resistance among *Shigella* has occurred since the 1940s when sulphonamide resistance among *Shigella* was first recognized in Japan (46). Since then, resistance to sulphonamide and other drugs has been increasing worldwide due to the excessive use of antimicrobial agents and failure to prevent the spread of multidrug-resistant strains of *Shigella*. This changing pattern of antimicrobial susceptibili-ty among *Shigella* serogroups poses a major problem in the determination of an appropriate drug for the treatment of *Shigella*-associated infections. *S. sonnei* was significantly more resistant to common antimicrobial agents, mainly tetracycline and co-trimoxazole, than were the other *Shigella* serogroups. These data need to be emphasized because *S. sonnei* is the predominant serogroup not only in this country but in the USA and other developed countries as well. The highest resistance to ampicillin was noted among the other *Shigella* serogroups (48-100%). While this is important to note, these isolates comprise less than 6% of the total *Shigella* serogroups recovered from stools. Aztreonam, gentamicin and the fluoroquinolone, ciprofloxacin, are the three drugs that show the greatest efficacy against all serogroups of *Shigella* in this study. Most cases of shigellosis occur in children aged less than 10 years. Of these three drugs, only ciprofloxacin is available for oral therapy, but paediatric use is limited by concerns about arthopathy and chrondrotoxicity. However, reports suggest that the fluoroquinolones are generally safe for the treatment of shigellosis in children (47,48).

The present study demonstrated that *S. sonnei* remained the predominant serogroup in Trinidad for the past 10 years. The data also showed that, although resistant strains exist among all serogroups, this resistance was relatively low and that most drugs are still efficacious in the empirical treatment of shigellosis, particularly ciprofloxacin, aztreonam, gentamicin, chloramphenicol, and trimethoprim-sulphamethoxazole. These findings confirm the need to formulate long-term surveillance programmes that would identify changes in antimicrobial susceptibility patterns and the dissemination of such information to clinicians.

## ACKNOWLEDGEMENTS

The author is grateful to the staff of the microbiology laboratory of the SFGH for providing the data.
